# Predicting the future direction of cell movement with convolutional neural networks

**DOI:** 10.1371/journal.pone.0221245

**Published:** 2019-09-04

**Authors:** Shori Nishimoto, Yuta Tokuoka, Takahiro G. Yamada, Noriko F. Hiroi, Akira Funahashi

**Affiliations:** 1 Department of Biosciences and Informatics, Keio University, Yokohama-shi, Kanagawa, Japan; 2 Faculty of Pharmacy, Sanyo-Onoda City University, Sanyo-Onoda, Yamaguchi, Japan; University of California Riverside, UNITED STATES

## Abstract

Image-based deep learning systems, such as convolutional neural networks (CNNs), have recently been applied to cell classification, producing impressive results; however, application of CNNs has been confined to classification of the current cell state from the image. Here, we focused on cell movement where current and/or past cell shape can influence the future cell movement. We demonstrate that CNNs prospectively predicted the future direction of cell movement with high accuracy from a single image patch of a cell at a certain time. Furthermore, by visualizing the image features that were learned by the CNNs, we could identify morphological features, e.g., the protrusions and trailing edge that have been experimentally reported to determine the direction of cell movement. Our results indicate that CNNs have the potential to predict the future direction of cell movement from current cell shape, and can be used to automatically identify those morphological features that influence future cell movement.

## Introduction

Recent advances in microscope automation have enabled the acquisition of large numbers of bioimages. Several approaches to analyzing these images are proposed. One successful approach is machine learning, which has been used primarily in cell classification [1]. Cell classification using conventional machine learning proceeds in two steps. Firstly, hand-crafted image features are extracted for each cell from the image (e.g., using Scale-Invariant Feature Transform [2], Histograms of Oriented Gradients [3], or CellProfiler [4]). Secondly, these features are used to train a classification model (e.g., Support Vector Machine, SVM [5], and Random Forest, RF [6]). As a result, the performance of these classifiers relies heavily on the appropriateness of the hand-crafted features chosen empirically. Moreover, because feature extraction and classifier training are independent of each other, they cannot work together to identify and use discriminative information maximally.

In recent years, deep learning methods have been used to overcome this limitation of conventional machine learning methods. Deep learning methods, especially convolutional neural networks (CNNs), automatically learn feature representations from the raw pixels of cell images. Therefore, CNNs can avoid using hand-crafted features. Furthermore, CNNs are jointly optimized with these feature representations to predict the class for each cell image. For general visual recognition tasks, CNNs have substantially outperformed conventional machine learning methods with hand-crafted features [7, 8], and they have been applied successfully to biological imaging [9, 10]. In cell classification, use of CNNs has produced impressive results [11–17]: e.g., the classification of abnormal morphology in MFC-7 breast cancer cells [13], the classification of cervical cells in cytology images [15]; the identification of malaria-infected cells [16]; and the automatic classification of Hep-2 (human epithelial-2) cell staining patterns [17].

The above applications of CNNs have focused on classification of the current cell state from the image. However, recent studies have demonstrated that the current and/or past cell shape influences the future cell state even after the original shape is lost. Akamura et al. (2016) demonstrated that the shape of a V2 neural progenitor cell affects the stochastic fate decision-making of the daughter cells even after the V2 cell loses the original geometry through mitotic rounding and division [18]. Kozawa et al. (2016) used Bayesian inference to predict cell division-timing based on progressive cell-shape changes [19]. Therefore, an interesting question is whether CNNs can be used to predict future cell state based on current and/or past cell shape.

Here, we focused on dynamic cell movement as a model system of cell shape influencing future cell movement. In general, cell movement can be conceptualized as a cyclic process [20]. The cell movement cycle begins with the formation of protrusions by actin polymerization. Protrusions are stabilized by adhering to the extracellular matrix (ECM). These adhesions serve as traction sites for the movement as the cell moves forward over them. At the cell rear, the adhesions with ECM are disassembled. Then the cell rear, the trailing edge, contracts mainly due to the pulling force generated by myosin II. As a result, the cell moves towards the protrusions. The motility and shape of individual migrating cells are closely related [21]. Jiang et al. (2005) demonstrated that the polarity of cell shape, i.e., wide front and a narrow rear, biases the direction of cell movement (i.e., moving direction) [22]. Ghosh et al. (2004) demonstrated that protrusions formed locally by actin polymerization define the moving direction [23]. We therefore hypothesized that CNNs can learn which morphological features influence moving direction and thus predict the future moving direction from cell shape at a certain time.

In general, it is not clear how and why CNNs arrive at a particular prediction decision, although several groups have attempted to interpret CNN predictions and propose possible methods for explaining CNNs decisions [24–27]. These methods can visualize the features of the image that contribute to the CNN predictions. Application of these methods is expected to identify morphological features that influence moving direction.

Here, we demonstrate that CNNs prospectively predict the future direction of cell movement with high accuracy from a single image patch of a cell at a certain time. Furthermore, to reveal how and why CNN models can predict future moving direction, we visualized the features of the cell images that were learned by the CNN models and contributed to their prediction: e.g., the protrusions and trailing edge. If the current morphology of cells can influence their future state, CNNs can predict the future state by determining cell morphology. Such an ability of CNNs should be particularly useful in those fields where prediction is crucial, such as the research on cell migration mechanism(s) or development of cell tracking systems.

## Results

### Training and validation of CNNs for predicting the future direction of cell movement

To illustrate that CNNs can predict cell state based on current cell shape, we set out to construct a CNN model that predicts the future direction of cell movement from a single image patch of a cell at a certain time. Firstly, we prepared image datasets from time-lapse phase-contrast microscopic images of migrating NIH/3T3 cells (see Materials and methods). We manually tracked the positions of the migrating cells. Then, each cell in each frame was annotated with the moving direction: i.e., toward upper right, upper left, lower left, or lower right. The annotation was determined based on the displacement at the time the net displacement exceeded the average diameter of NIH/3T3 cells (18 *μ*m, http://bionumbers.hms.harvard.edu/bionumber.aspx?id=108905). For each annotated cell in each frame, we created image patches of 128 × 128 pixels centered on its position coordinate. The NIH/3T3 dataset comprised 785 image patches for 40 cells (Table 1). We separated the NIH/3T3 dataset into the training, validation, and testing datasets. The training and validation datasets were used in training by cross-validation, and the testing dataset was used in the evaluation of prediction.

**Table 1:** Number of image patches per moving direction.

Cell Type	Moving Direction	train	validation	test	total
NIH/3T3	upper right	107	36	36	179
	upper left	112	38	38	188
	lower left	145	49	48	242
	lower right	105	36	35	176
U373	upper right	99	33	33	165
	upper left	151	51	51	253
	lower left	57	20	19	96
	lower right	168	57	56	281
hTERT-RPE1	upper right	57	20	19	96
	upper left	438	146	147	731
	lower left	162	55	54	271
	lower right	141	47	47	235

**Fig 1:**
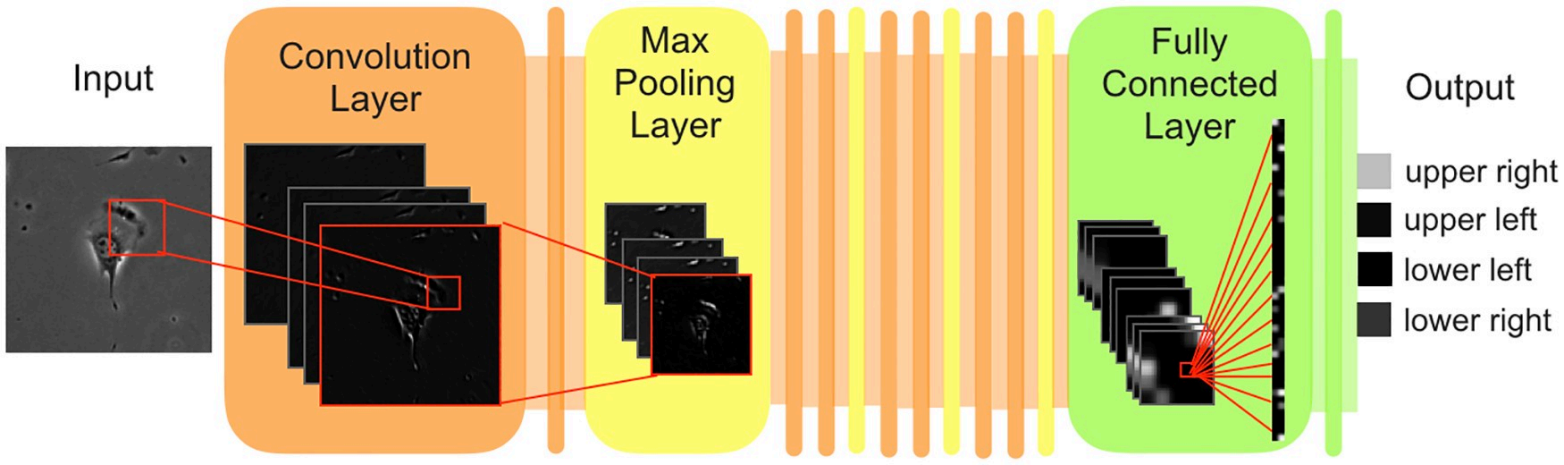
Architecture of CNN models for predicting the future direction of cell movement. In the flowchart, an image patch of a NIH/3T3 cell annotated on the upper right (Input) is presented to a CNN model. The input is processed through a series of repeating convolutional layers (orange) and max-pooling layers (yellow). In the convolutional layer, the activation images illustrate extracted feature maps of the sample image patch (Input). The red boxes and lines illustrate the connections within the CNN model. After repeating processing through convolutional layers and max-pooling layers, fully connected layers are used for prediction (green). The network output (Output) represents the distribution over four moving directions.

We used these datasets to train and test CNN model for predicting the direction of cell movement. In our CNN model, input image patches are processed through multiple convolutional layers and max-pooling layers. In convolutional layers, trainable sets of filters are applied at different spatial locations using a stride of a certain size, thereby extracting features associated with moving direction as feature maps. In max-pooling layers, feature maps are down-sampled by computing the maximum value of a feature map over a region, which reduces variance and increases translational invariance [28]. After repeating the processing through convolutional layers and max-pooling layers, two fully connected layers are used for prediction. Our CNN model arrange 14 layers into eight convolutional layers, four max-pooling layers, and two fully connected layers, consisting of 162,704 trainable parameters in total (more detail in Material and Methods, network architecture shown in Fig 1 and Table 2). This model was optimized by Bayesian optimization based on VGG-16 [29] using the training and validation NIH/3T3 datasets.

**Table 2:** CNN model-relevant hyperparameters.

Layer	Type	Description
1	Convolution	Filter size = 5 × 5, Number of filters = 8, Stride size = 1
2	Convolution	Filter size = 5 × 5, Number of filters = 32, Stride size = 1
3	Max-pooling	Filter size = 2 × 2, Stride size = 2
4	Convolution	Filter size = 3 × 3, Number of filters = 40, Stride size = 1
5	Convolution	Filter size = 5 × 5, Number of filters = 32, Stride size = 1
6	Max-pooling	Filter size = 2 × 2, Stride size = 2
7	Convolution	Filter size = 5 × 5, Number of filters = 48, Stride size = 1
8	Convolution	Filter size = 5 × 5, Number of filters = 64, Stride size = 1
9	Max-pooling	Filter size = 2 × 2, Stride size = 2
10	Convolution	Filter size = 5 × 5, Number of filters = 64, Stride size = 1
11	Convolution	Filter size = 5 × 5, Number of filters = 72, Stride size = 1
12	Max-pooling	Filter size = 2 × 2, Stride size = 2
13	Fully connected	Number of neurons = 1000
14	Fully connected	Number of neurons = 4

To assess the generalization performance of our CNN model, we trained them in 4-fold cross-validation using the training and validation NIH/3T3 datasets and tested them using the testing NIH/3T3 dataset. The testing dataset was evaluated by using the model with the highest accuracy in cross-validation. We evaluated the performance of trained CNN model by calculating the average classification accuracy (ACA) and the mean class accuracy (MCA) (see Materials and methods). While the expected value of both ACA and MCA, if the data are randomly predicted, is 25%, our CNN model achieved a high ACA of 87.89% and a high MCA of 87.87% with the testing NIH/3T3 dataset. Prediction accuracy was not affected by analyzing cells with differences in migration speed (S1(A) Fig). To evaluate prediction errors, we created a confusion matrix (S2(A) Fig). This matrix showed that our CNN model predicted the direction adjacent to the true direction as misprediction in many cases. On the other hand, the number of mispredictions for the reverse direction was considerably lower. For visual clarity, we overlaid the predicted directions on raw time-series images (S1 Movie).

Next, we compared our model with conventional methods (SVM, nonlinear SVM, and RF). These methods were trained using the same training NIH/3T3 dataset, and their prediction accuracy was evaluated using the testing dataset. The MCA was 41.46% for SVM, 44.77% for nonlinear SVM, and 64.42% for RF.

We also evaluated whether our model was applicable to other cell types. For U373 and hTERT-RPE1 cells, we created image patches of 128 × 128 pixels centered on its position coordinate, similar to that for the NIH/3T3 dataset. The U373 dataset comprised 795 image patches for 12 cells, and the hTERT-RPE1 dataset comprised 1333 image patches for 20 cells (Table 1). We also separated the U373 and hTERT-RPE1 datasets into the training, validation, and testing datasets. We trained the model in 4-fold cross-validation using the training and validation U373 and hTERT-RPE1 datasets and tested them using the same procedure as for the NIH/3T3 dataset. Our CNN model achieved high ACA (U373, 81.76%; hTERT-RPE1, 95.13%) and high MCA (U373, 80.65%; hTERT-RPE1, 94.22%). As in the analysis of NIH/3T3 cell migration, these prediction accuracies were not affected by the speed of migration (S1(B) and S1(C) Fig). The confusion matrices (S2(B) and S2(C) Fig) showed that our CNN model predicted the direction adjacent to the true direction as misprediction in many cases. For visual clarity, we also overlaid the predicted directions on the raw time-series images (S2 and S3 Movies).

### Visualizing image features learned by the CNN model

**Fig 2:**
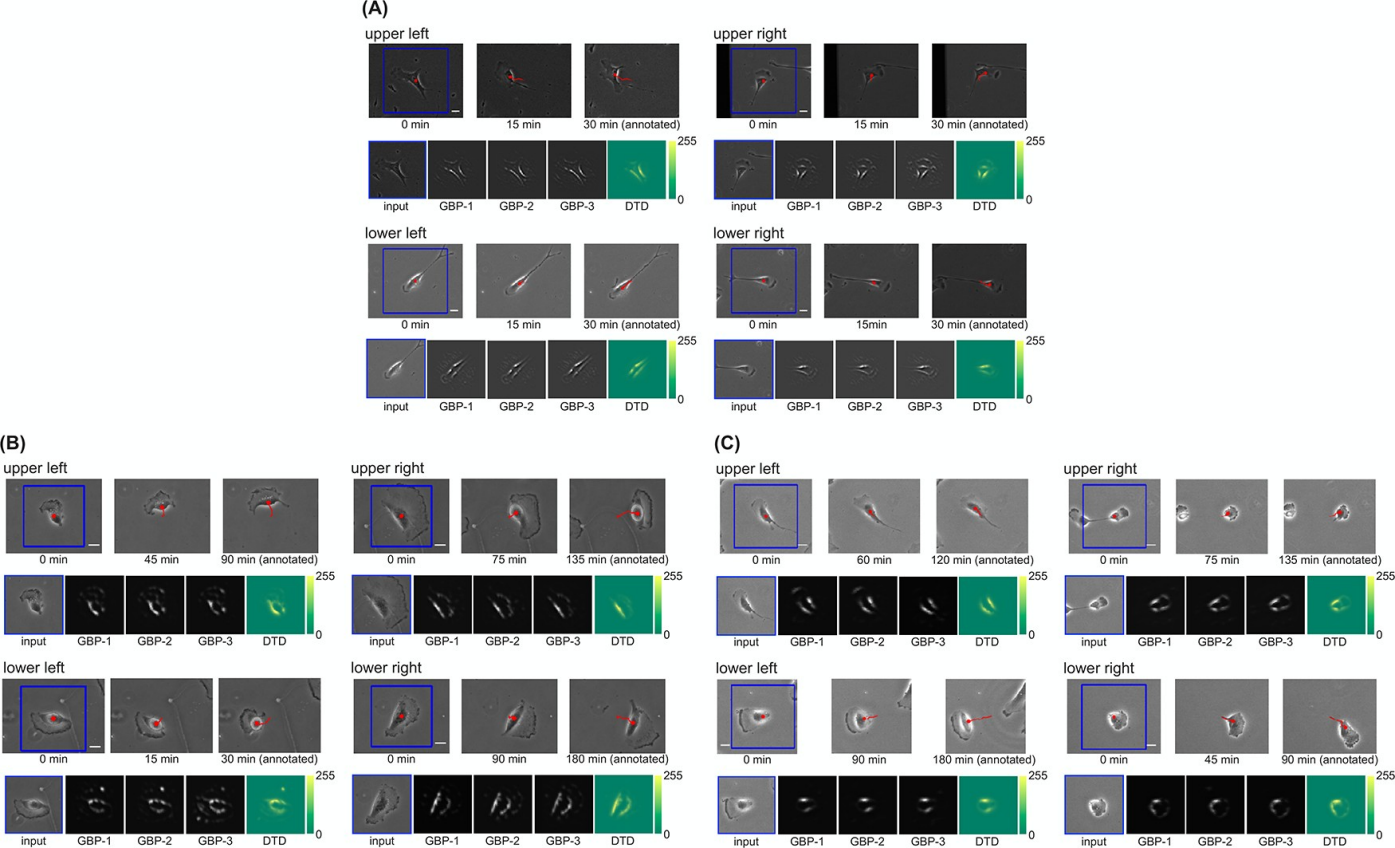
Visualized image features learned by the CNN models. (A) NIH/3T3 dataset. (B) U373 dataset. (C) hTERT-RPE1 dataset. For each moving direction, each group of images shows exemplary results (i.e., those for a correctly predicted test image patch). The upper row of each group of images comprises—from left to right—the frame corresponding to the input image patch, the frame imaged in the middle between the left frame and the right frame, and the frame when the moving direction was annotated (scale bars, 20 *μ*m). The time under each frame shows the elapsed time since the leftmost frame was imaged. The blue bounding box indicates the area corresponding to the input image patch. The red dot indicates the position of the cell obtained by manual tracking. The red line indicates the trajectory of cell movement starting from the position of the cell at 0 min. The lower row of each group of images comprises—from left to right—the input image patch, the local features visualized by GBP for the feature maps whose maximum activations were top three, and the heatmap of pixel-wise relevance calculated by DTD.

Next, to identify the morphological features influential in the prediction of our CNN model, we quantified and visualized the image features learned by our CNN model. Firstly, we used guided backpropagation (GBP) [27], which visualizes the local feature of the input image that most strongly activates a CNN’s particular neuron (see Material and methods). For each dataset, we presented each test image to the trained CNN model. Then, GBP was applied to a single maximum activation in each feature map of the last convolutional layer, producing local features of the input image patch. As a result, local features corresponding to the protrusion and the trailing edge contracting in the moving direction were identified for all of the NIH/3T3, U373, and hTERT-RPE1 datasets (lower parts of each image group, Fig 2). Next, we quantified and visualized global features of the image patches contributing to the CNN prediction of the moving direction by using deep Taylor decomposition (DTD) [26]. DTD quantifies the degree of contribution to the CNN prediction (relevance) of the input image patch (see Materials and methods) on a pixel-by-pixel basis. For each dataset, we presented each test image to the trained CNN model. Then, by applying DTD to the prediction result, pixel-wise relevances were calculated. We visualized these pixel-wise relevances as heatmaps (Fig 2, lower right). For a part of the NIH/3T3, U373, and hTERT-RPE1 datasets, pixels corresponding to the protrusion and trailing edge simultaneously contributed to the prediction of the CNN model (Fig 2(A) upper right, Fig 2(B) lower right, Fig 2(C) lower right, S4 and S5 Movies). In addition, pixels corresponding to halos generated in phase-contrast microscopic images contributed to the prediction of the CNN model (Fig 2(A) upper left, lower left, lower right, Fig 2(B) upper left, upper right, lower left, Fig 2(C) upper left, upper right, lower left). We confirmed that these results were also consistent with the results of simple visualization with occlusion [24] (S3 Fig).

## Discussion

Previous applications of CNNs to cell classification have focused on classification of the current cell state from an image [11–17]. In contrast, here, we focused on images of dynamic cell movement and demonstrated that CNNs can prospectively predict the future direction of cell movement with high accuracy.

Our model showed strong performance because it was optimized by Bayesian optimization. This optimization targeted dropout and data augmentation, but we did not investigate their relative contributions to accuracy improvement. In ablation experiments with the NIH/3T3 dataset, MCA was 87.87% with both processes, 87.85% with the ablated dropout, and 85.73% with the ablated augmentation. We concluded that data augmentation contributed more to accuracy than dropout.

Our model can predict the direction of cell migration with high accuracy, yet there is room for improvement of the training datasets used for our model. Generally, increasing the size of a training dataset improves accuracy, but we could not easily increase the size of our training dataset because its creation required a lot of manual work in segmentation to identify cell location and tracking to determine the direction of cell movement. Recent studies suggested a fully automatic algorithm for segmentation and tracking [30, 31]; this algorithm would eliminate the need for manual dataset creation and greatly increase the accuracy.

We trained SVM and RF using the designed features (S1 Table). The accuracies of SVM and RF were lower than that of CNNs, which can automatically extract optimal features. The accuracies of SVM and RF may be improved if we could select an appropriate feature.

Average speed and directionality are important parameters that define cell motility [32]. Each of these parameters differed significantly among the NIH/3T3, U373, and hTERT-RPE1 datasets (S4 Fig). The apparent morphology of migrating cells also differed qualitatively among the three datasets: for example, protrusions were more prominent and broad in U373 cells than in NIH/3T3 and hTERT-RPE1 cells (Fig 2 and S1–S3 Movies). Despite these differences, our CNN model achieved the same degree of prediction accuracy for all three cell types, perhaps because they could learn the morphological features, such as the protrusions and the trailing edge (Fig 2), which are the characteristic features of cell movement regardless of cell type or substrate, except in some specific cell types [20, 33].

Because cell classification using conventional machine learning is built upon the extraction of hand-crafted features [1–4], the features affecting the classification result depend on the choice of hand-crafted features. These hand-crafted features are abstract representations of the cell image and are therefore diffcult to intuitively relate to visual inspection of the cell image. In contrast, because CNNs automatically and jointly learn optimal feature representations and the prediction task directly from the raw pixels of the cell image, the features affecting the prediction result do not depend on the feature selection and are optimized for the prediction. Furthermore, because these features can be visualized on the cell image by using the approach used in this study, they can be intuitively related to visual inspection of the cell image. Here, we identified morphological features, such as protrusions and trailing edge, which have been shown experimentally to influence the moving direction from some images[for review, see [20]]: for instance, protrusions generated locally by actin polymerization determine the moving direction [23], and the position of the trailing edge influences the moving direction via stress fiber organization [34]. In addition, a halo was shown to be a learned features in some parts of images. Our results indicate that such important morphological features that can influence cell movement can be identified automatically, by using CNNs to predict cell movement and then visualizing the image feature(s) contributing to this prediction. On the other hand, there are still deficiencies in our feature analysis. It focuses on the characteristic features that are detectable visually, such as the protrusions and trailing edge, and thus is not comprehensive. Therefore, our analysis may have failed to take into account some other important features. In the future, we need to identify unknown image features without prior biological knowledge via quantitative analysis (e.g., unsupervised clustering).

A halo is formed due to the thickness of cells and the refractive index of the composition. Previous research postulated that a halo was an obstacle in tracking analysis and removed the halo region from the image for tracking analysis [35]. In our results, however, CNN regarded the halo as important information in predicting the migration direction. This result implies that the correct way of analyzing halo could improve the tracking analysis.

Cell movement consisted of two steps: (1) movement by the adhesion of a protrusion to the ECM forward over the cell body and (2) movement by pulling force generated by myosin II with the disassembly of the ECM at the cell rear. The former takes about 3 min or less [36] and the latter about 6 min [37], but each image was acquired for 15 min. Our model predicted the migration direction in the next frame from the previous frame. Therefore, the time resolution of our dataset was too low to identify these steps separately, and higher time resolution (observation for <3 min) would be needed.

In conclusion, we focused on dynamic cell movement as a model system in which cell shape influences cell state. Our findings indicate that use of CNNs can enable not only the prediction of the future cell state but also the identification of morphological features that can influence the future cell movement. Based on these findings, we believe that our approach could be applied to the analysis of other cell mechanisms in which cell shape can influence future processes, such as cell division [19] and differentiation [38]. Our results are also useful for development of an automatic tracking system. Although two-dimensional analysis was sufficient for the target cells in our study, three-dimensional analysis is necessary when targeting tissue cells. Analysis of the real cell state would require three-dimensional data, which could reveal unknown biological phenomena.

## Materials and methods

### Time-lapse phase-contrast microscopic images

To create datasets for training our CNN model, we prepared time-lapse phase-contrast microscopic images of cell movement in different ways for each dataset; details are provided below.

#### NIH/3T3 dataset

NIH/3T3 fibroblasts were plated on glass-based dishes precoated with 5 *μ*g/cm^2^ fibronectin at a density of 500 cells/cm^2^. After overnight incubation, cell movements were monitored with an inverted microscope (IX81, Olympus) equipped with an on-stage incubation chamber that maintained the temperature at 37°C and the CO_2_ concentration at 5%, using a 20× objective (0.45 numerical aperture). Phase-contrast images were collected with CCD video cameras (ORCA-Flash4.0 or ORCA-R2; Hamamatsu) at 5-min intervals, digitized and stored as image stacks by using Micro-Manager software (Open Imaging).

#### U373 dataset

Time-lapse phase-contrast microscopic images of glioblastoma–astrocytoma U373 cells were obtained from the dataset used in the ISBI (International Symposium on Biomedical Imaging) cell tracking challenge 2015 [39, 40]. U373 cells moving on a polyacrylamide substrate were monitored with a microscope (Nikon) using a 20× objective (0.5 numerical aperture), and phase-contrast images were acquired at 15-min intervals.

#### hTERT-RPE1 dataset

Time-lapse phase-contrast microscopic images of telomerase-immortalized human retinal pigment epithelium (hTERT-RPE1) cells [41] were obtained from the Cell Image Library (http://flagella.crbs.ucsd.edu/home). Movement of U373 cells on glass coverslips in DMEM/F12 medium containing 10% FBS, glutamine, HEPES, penicillin, and streptomycin was observed under a microscope (Leica DMIRE2; 10× objective/0.3 numerical aperture). Phase-contrast images were acquired at 3-min intervals.

### Annotation of the moving direction

**Fig 3:**
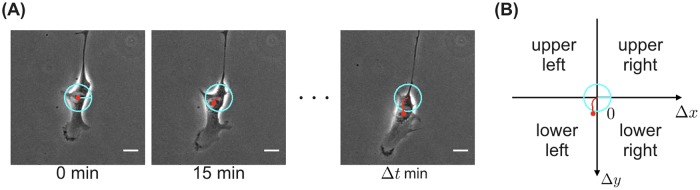
Annotation of the moving direction. (A) Exemplary time-lapse images of a migrating cell (scale bars, 20 *μ*m). The time under each frame shows the elapsed time since the leftmost frame (annotation target) was imaged. Δ*t* is the time when the net displacement $\sqrt{\Delta x^{2}+\Delta y^{2}}$ exceeded the average diameter of NIH/3T3 cells. The red dot indicates the position of the cell obtained by manual tracking. The red line indicates the trajectory of cell movement starting from the position of the cell at 0 min. The radius of the cyan circle is the average diameter of NIH/3T3 cells. (B) Annotating one of the four moving directions. According to the value of cell displacement (Δ*x*, Δ*y*) at the time Δ*t*, the moving direction was annotated as shown in the figure. The red line and the cyan circle are the same as those of the frame at Δ*t* min in (A).

For each dataset, we manually tracked the positions of migrating cells by using the Manual Tracking plugin of ImageJ (National Institutes of Health). For each cell in each frame, we annotated one of the four moving directions according to the value of displacement (Δ*x*, Δ*y*) at the time the net displacement $\sqrt{\Delta x^{2}+\Delta y^{2}}$ (the distance between the initial and final positions) exceeded the average diameter of NIH/3T3 cells (18 *μ*m (http://bionumbers.hms.harvard.edu/bionumber.aspx?id=108905)) (Fig 3). The four moving directions were defined as follows: (i) Δ*x* ≥ 0, Δ*y* ≤ 0 = upper right; (ii) Δ*x* < 0, Δ*y* ≤ 0 = upper left; (iii) Δ*x* < 0, Δ*y* > 0 = lower left; and (iv) Δ*x* ≥ 0, Δ*y* > 0 = lower right. Cells whose net migration did not exceed their diameter were excluded from the datasets. In the U373 and hTERT-RPE1 datasets, the net displacement was evaluated as in the NIH/3T3 dataset, but at 15-min intervals.

For each annotated cell, we cropped the image to a bounding box of 256 × 256 pixels for the NIH/3T3 dataset, 170 × 170 pixels for the U373 dataset, and 220 × 220 pixels for the hTERT-RPE1 dataset, centered on the cell *x*, *y* coordinates. Cropped image patches were resized to 128 × 128 pixels for each dataset.

### CNNs for predicting the future direction of cell movement

To prospectively predict the future direction of cell movement from a single image patch of a cell at a certain time, we used CNN models [42, 43] with the architecture shown in Fig 1 and Table 2. Our CNN model outputs the migration direction in the next frame, using the image before the previous frame as input. Each CNN model comprised consecutive convolutional layers, max-pooling layers, and fully connected layers. The last fully connected layer consisted of four neurons, with each neuron corresponding to the future direction of cell movement. We applied the softmax function to the activations of the last fully connected layer to produce a distribution over four moving directions. Each convolutional layer and the first fully connected layer was followed by a nonlinear activation function. We used rectified linear units, which have been shown to introduce nonlinearities without suffering from the vanishing gradient problem [44]. From the point of view of architecture design, selecting ReLU in a deep neural network–based model reportedly shows the best performance [45]. To enable the CNN model to predict the future moving direction for image patches with various intensity values, the pixel values of each input image patch were normalized to within the range of 0 to 1 by first subtracting the minimum intensity value of the image patch, and then dividing by the maximum intensity value of the resulting image patch [17].

### Training and validation of CNN model

We trained the CNN model in 4-fold cross-validation. At each fold, we used a softmax loss function and trained the CNN model for 50 epochs, using stochastic gradient descent with stratified batches of 46 images. To reduce the influence of imbalanced moving directions in the datasets, we multiplied the loss function by the class weight expressed by the following equation:

$$\begin{array}{c} \frac{\sum_{k}^{n}N_{k}}{nN_{k}} \end{array}$$

where *n* is the number of classes (moving directions), and *N*_*k*_ is the number of training data of class *k*. We initialized all weights in a CNN model by using the HeNormal algorithm, which sets the weights of each layer according to a scaled Gaussian distribution whose standard deviation corresponds to the input size of each layer [46]. We used standard values for the base learning rate (0.021) and momentum (0.5). For each epoch, we evaluated the prediction accuracy of a CNN model using test data. The evaluation criteria were the ACA and the MCA: ACA is the overall correct prediction rate of all test data, and MCA is the average of the per-class accuracies defined as follows:

$$\begin{array}{c} \text{MCA}=\frac{1}{n}\sum_{k=1}^{n}{\text{CCR}}_{k} \end{array}$$

where CCR_*k*_ is the prediction accuracy of class (moving direction) *k* and *n* is the number of classes. To prevent the CNN models from overfitting to training data, we (a) trained the models using vertical and horizontal reflections of the image patches as well as rotations by 90°, 180°, and 270°, in addition to the original training data; and (b) applied dropout [47] to the first fully connected layer (dropout rate, 0.3). The structure of the network is based on VGG-16 [29], and parameter tuning suitable for the dataset was performed by Bayesian optimization in SigOpt (https://sigopt.com). SigOpt was used as the optimization platform. For each fold, we used the best score of each criterion for 50 epochs as the prediction accuracy of the models.

For the following analysis of image features learned by our CNN model, we used this model with the best MCA in cross-validation for the test data. We implemented and trained the CNN model by using Chainer [48], which is open-source software for machine learning.

### Training of conventional methods for predicting the direction of cell movement

We trained three conventional methods (SVM, nonlinear SVM, and RF) for predicting the direction of cell movement in 4-fold cross-validation and tested them using the same datasets as for our CNN model. The input was a 52-dimensional feature vector (e.g., texture angular, contrast, and entropy) extracted from the input image using CellProfiler [4]. All feature vectors were input to each conventional method without pre-processing. The kernel of SVM used “linear,” and the kernel of nonlinear SVM used “rbf.” The criterion of RF used “gini.”

### Visualizing image features learned by CNN models

We quantified and visualized features learned by the CNN model as described in the Results. GBP [27] was used to visualize local features of input image patches specific to the annotated moving direction. In this method, an input image is presented to a CNN, and high-level feature maps are computed throughout the layers. Typically, a single neuron activation is left non-zero in the high-level feature map. The single non-zero activation is back-propagated to input pixel space. Finally, the part of the input image that is most strongly activating the single non-zero neuron (i.e., the part that is most discriminative) is reconstructed. For each dataset, we presented each test image patch to trained CNN model, and a single maximum activation in each feature map of the last convolutional layer was back-propagated to input pixel-space, resulting in producing local features of the input image.

To quantify and visualize global features of input image patches contributing to the CNN prediction of moving direction, we used DTD [26], which can be summarized as follows. As a premise, each neuron of a CNN is viewed as a function that can be expanded and decomposed on its input variables. A CNN prediction of the moving direction is obtained by forward-propagation of input pixel values {*x*_*p*_} and is encoded by the output neuron *x*_*f*_. The output neuron is assigned a relevance score *R*_*f*_ = *x*_*f*_ representing the total evidence for the specific moving direction. Relevance is then decomposed and back-propagated from the top layer down to the input. As a result, the pixel-wise relevance (i.e., the degree of contribution to the CNN prediction) is calculated for each pixel of the input image. The pixel-wise relevances can be visualized as a heatmap. For each dataset, we presented each test image patch to trained CNN model, and DTD was applied to the CNN prediction output.

Using simple visualization with occlusion [24] for local regions of the input image that contributed to the movement prediction, we verified whether consistency with the morphological features obtained by GBP and DTD was maintained. An input image with masking around a certain pixel was presented to the CNN model, and the normalized negative output value [0, 255] of the class of the input image was defined as a contribution to the prediction in this pixel. By performing this occlusion operation pixel-wise, we created a contribution map for prediction of an input image. We set the mask size to 7 × 7 pixels.

## Supporting information

S1 Fig Accuracy of cell movement prediction by the CNN model using testing datasets and the number of cells at each speed. (A) NIH/3T3 dataset. (B) U373 dataset. (C) hTERT-RPE1 dataset.
(TIF)

S2 Fig Confusion matrices for the prediction of four directions by our CNN model using testing datasets. (A) NIH/3T3 dataset. (B) U373 dataset. (C) hTERT-RPE1 dataset.
(TIF)

S3 Fig Image features visualized with occlusion. (A) NIH/3T3 dataset. (B) U373 dataset. (C) hTERT-RPE1 dataset. For each movement direction, each group of images shows typical results for a correctly predicted test image patch. Scale bars, 20 *μm*.
(TIF)

S4 Fig Motility values calculated for the NIH/3T3, U373, and hTERT-RPE1 datasets. (A) Boxplot of the average speed of the cell, *v*. (B) Boxplot of the directionality of the cell, *k* (*n* = 785 cell images in the NIH/3T3 dataset, 795 cell images in the U373 dataset, and 1,333 cell images in the hTERT-RPE1 dataset). P-value is from two-sided Mann-Whitney rank test. For each image in the datasets, we first measured the time required until the net displacement Δ*r* exceeded the average diameter of NIH/3T3 cells, which is the time interval Δ*t* until the moving direction was annotated. Then, we calculated the total distance ∑Δ*d* traveled by the cell in the time interval Δ*t*. Regarding the NIH/3T3 and hTERT-RPE1 dataset, the net displacement Δ*r* and movement distance Δ*d* were calculated at 15-min intervals according to the shooting interval of the U373 dataset. The average speed was calculated by the equation *v* = ∑Δ*d*/Δ*t* [22]. The directionality was calculated by dividing the net displacement Δ*r* by the total distance ∑Δ*d* [22]. The directionality was used to measure how often the cell tended to turn. Cells that frequently make turns will yield a *k* value close to 0, whereas cells that persistently move along one direction will yield a *k* value close to 1.
(TIF)

S1 Table Features extracted by CellProfiler. (CSV)

S1 Movie Time-lapse phase-contrast images of migrating NIH/3T3 cells overlaid with prediction results (MP4). Boxes show the size of the patch used for prediction. Color of the boxes corresponds to the predicted direction: red, upper right; blue, upper left; green, lower left; yellow, lower right.
(MP4)

S2 Movie Time-lapse phase-contrast images of migrating U373 cells overlaid with prediction results (MP4). Boxes show the size of the patch used for prediction. Color of the boxes corresponds to the predicted direction: red, upper right; blue, upper left; green, lower left; yellow, lower right.
(MP4)

S3 Movie Time-lapse phase-contrast images of migrating hTERT-RPE1 cells overlaid with prediction results (MP4). Boxes show the size of the patch used for prediction. Color of the boxes corresponds to the predicted direction: red, upper right; blue, upper left; green, lower left; yellow, lower right.
(MP4)

S4 Movie Time-lapse phase-contrast images of migrating NIH/3T3 cells overlaid with prediction and GBP results (MP4). Boxes show the size of the patch used for prediction. Color of the boxes corresponds to the predicted direction: red, upper right; blue, upper left; green, lower left; yellow, lower right. The local features were visualized by GBP for the feature maps whose maximum activations were the top one.
(MP4)

S5 Movie Time-lapse phase-contrast images of migrating NIH/3T3 cells overlaid with prediction results and DTD results (MP4). Boxes show the size of the patch used for prediction. Color of the boxes corresponds to the predicted direction: red, upper right; blue, upper left; green, lower left; yellow, lower right. The heatmap of pixel-wise relevance calculated by DTD.
(MP4)
