# Supplementary figures and images for: Predicting the future direction of cell movement with convolutional neural networks
Source: PLoS One. 2019 Sep 4;14(9):e0221245. doi: 10.1371/journal.pone.0221245 (PMC6726366; doi:10.1371/journal.pone.0221245)

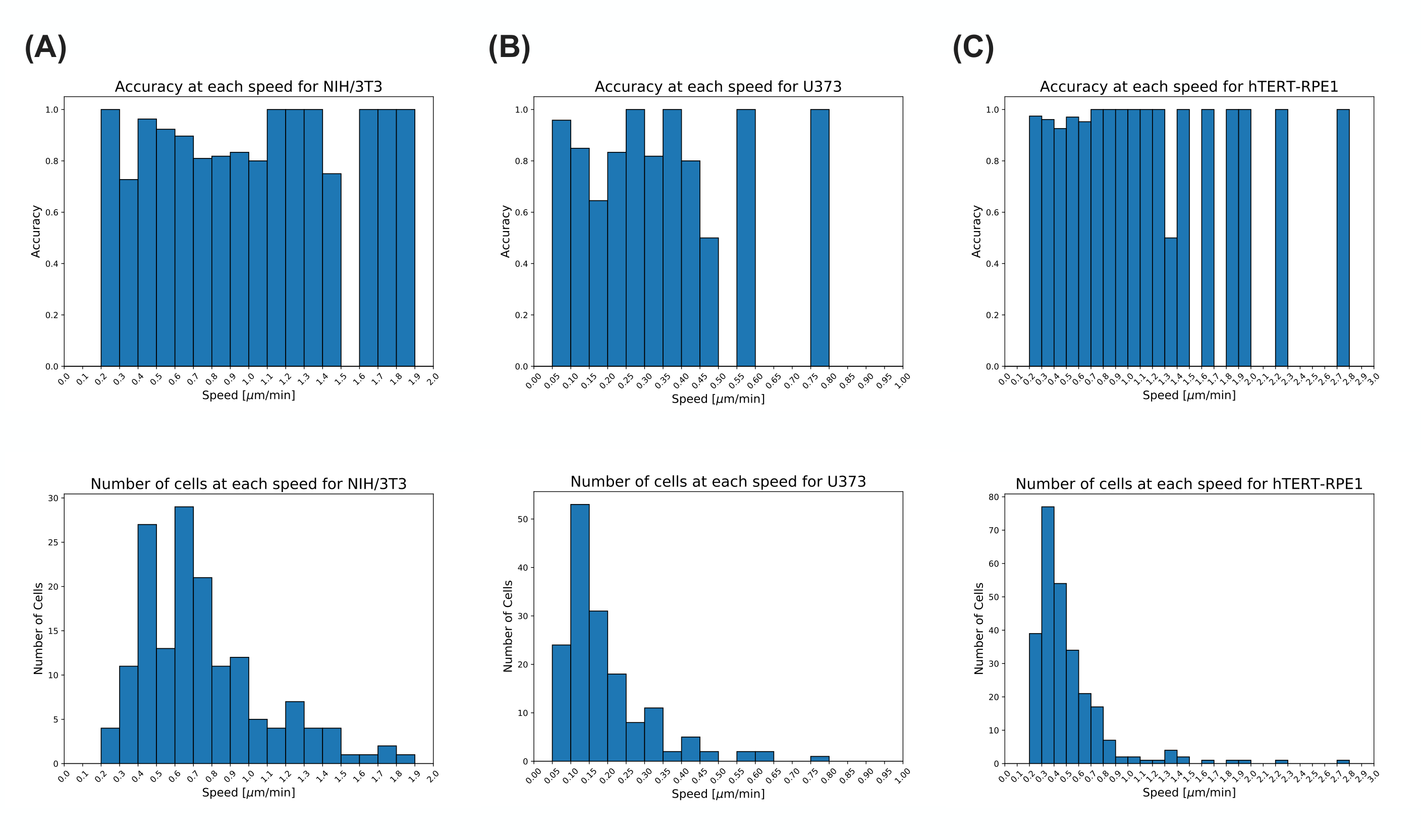

Supplement: S1 Fig — (A) NIH/3T3 dataset. (B) U373 dataset. (C) hTERT-RPE1 dataset. (TIF) [file pone.0221245.s001.tif]

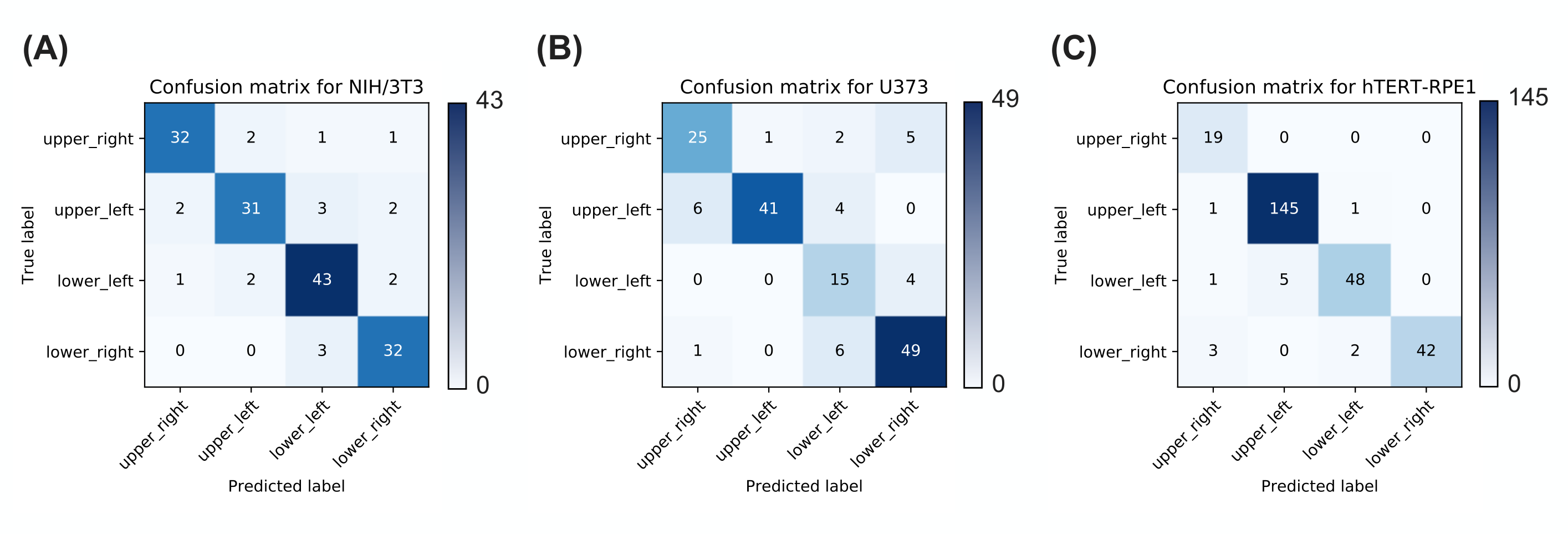

Supplement: S2 Fig — (A) NIH/3T3 dataset. (B) U373 dataset. (C) hTERT-RPE1 dataset. (TIF) [file pone.0221245.s002.tif]

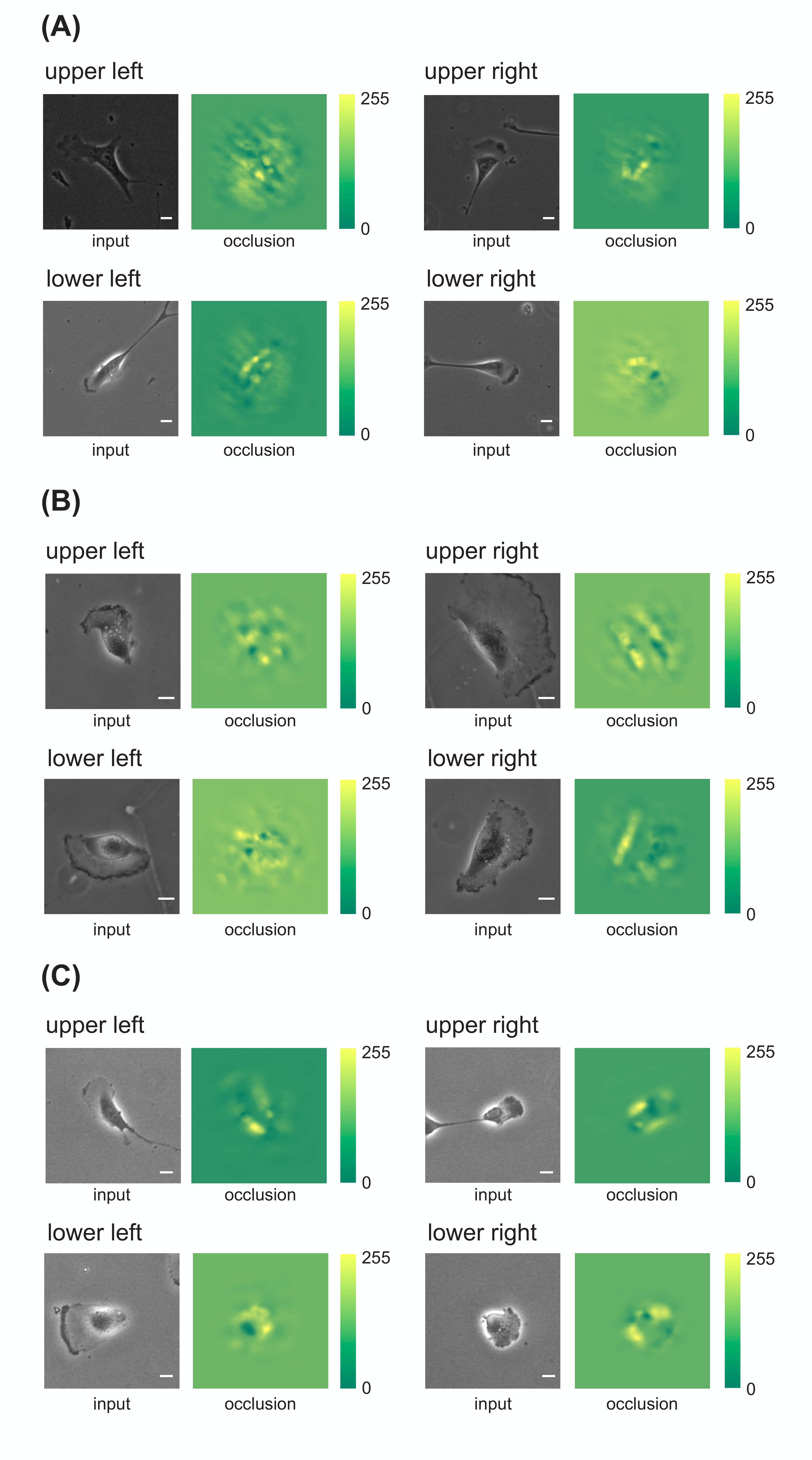

Supplement: S3 Fig — (A) NIH/3T3 dataset. (B) U373 dataset. (C) hTERT-RPE1 dataset. For each movement direction, each group of images shows typical results for a correctly predicted test image patch. Scale bars, 20 μm. (TIF) [file pone.0221245.s003.tif]

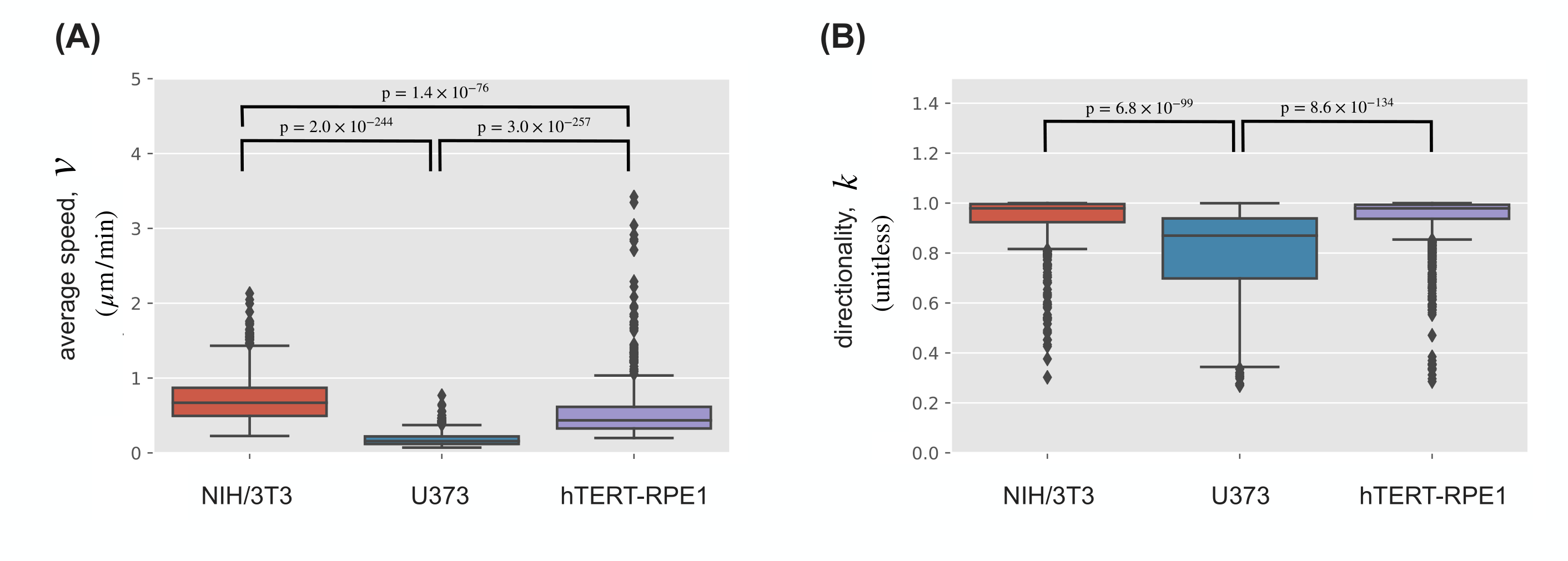

Supplement: S4 Fig — (A) Boxplot of the average speed of the cell, v. (B) Boxplot of the directionality of the cell, k (n = 785 cell images in the NIH/3T3 dataset, 795 cell images in the U373 dataset, and 1,333 cell images in the hTERT-RPE1 dataset). P-value is from two-sided Mann-Whitney rank test. For each image in the datasets, we first measured the time required until the net displacement Δr exceeded the average diameter of NIH/3T3 cells, which is the time interval Δt until the moving direction was annotated. Then, we calculated the total distance ∑Δd traveled by the cell in the time interval Δt. Regarding the NIH/3T3 and hTERT-RPE1 dataset, the net displacement Δr and movement distance Δd were calculated at 15-min intervals according to the shooting interval of the U373 dataset. The average speed was calculated by the equation v = ∑Δd/Δt [22]. The directionality was calculated by dividing the net displacement Δr by the total distance ∑Δd [22]. The directionality was used to measure how often the cell tended to turn. Cells that frequently make turns will yield a k value close to 0, whereas cells that persistently move along one direction will yield a k value close to 1. (TIF) [file pone.0221245.s004.tif]
